# A Biomechanical Comparison of Proportional Electromyography Control to Biological Torque Control Using a Powered Hip Exoskeleton

**DOI:** 10.3389/fbioe.2017.00037

**Published:** 2017-06-30

**Authors:** Aaron J. Young, Hannah Gannon, Daniel P. Ferris

**Affiliations:** ^1^Woodruff School of Mechanical Engineering, Georgia Institute of Technology, Atlanta, GA, United States; ^2^Department of Biomedical Engineering, University of Michigan, Ann Arbor, MI, United States; ^3^School of Kinesiology, University of Michigan, Ann Arbor, MI, United States

**Keywords:** robotic exoskeleton, powered orthosis, electromyography control, biomechanics of human walking, metabolic cost, exoskeleton control

## Abstract

**Background:**

Despite a large increase in robotic exoskeleton research, there are few studies that have examined human performance with different control strategies on the same exoskeleton device. Direct comparison studies are needed to determine how users respond to different types of control. The purpose of this study was to compare user performance using a robotic hip exoskeleton with two different controllers: a controller that targeted a biological hip torque profile and a proportional myoelectric controller.

**Methods:**

We tested both control approaches on 10 able-bodied subjects using a pneumatically powered hip exoskeleton. The state machine controller targeted a biological hip torque profile. The myoelectric controller used electromyography (EMG) of lower limb muscles to produce a proportional control signal for the hip exoskeleton. Each subject performed two 30-min exoskeleton walking trials (1.0 m/s) using each controller and a 10-min trial with the exoskeleton unpowered. During each trial, we measured subjects’ metabolic cost of walking, lower limb EMG profiles, and joint kinematics and kinetics (torques and powers) using a force treadmill and motion capture.

**Results:**

Compared to unassisted walking in the exoskeleton, myoelectric control significantly reduced metabolic cost by 13% (*p* = 0.005) and biological hip torque control reduced metabolic cost by 7% (*p* = 0.261). Subjects reduced muscle activity relative to the unpowered condition for a greater number of lower limb muscles using myoelectric control compared to the biological hip torque control. More subjects subjectively preferred the myoelectric controller to the biological hip torque control.

**Conclusion:**

Myoelectric control had more advantages (metabolic cost and muscle activity reduction) compared to a controller that targeted a biological torque profile for walking with a robotic hip exoskeleton. However, these results were obtained with a single exoskeleton device with specific control configurations while level walking at a single speed. Further testing on different exoskeleton hardware and with more varied experimental protocols, such as testing over multiple types of terrain, is needed to fully elucidate the potential benefits of myoelectric control for exoskeleton technology.

## Introduction

Engineers and scientists have been developing powered exoskeleton devices to augment normal human strength or aid individuals with disabilities since the 1960s (Laurence et al., 1969; Fick and Makinson, 1971; Vukobratovic et al., 1974; Zoss et al., 2006; Dollar and Herr, 2007, 2008; Yan et al., 2015). Today, there are dozens of robotic lower limb exoskeletons that have been created in academic or industry research laboratories. Some of the most visible exoskeletons include the DARPA-funded exoskeletons (Garcia et al., 2002; Chu et al., 2005; Guizzo and Goldstein, 2005; Walsh et al., 2006), the new wave of medical and rehabilitation lower limb exoskeletons (Zeilig et al., 2012; Kolakowsky-Hayner et al., 2013; Farris et al., 2014a), and industrial and/or military human performance augmentation exoskeletons (France’s Slender Hercule Exoskeleton Is No Lightweight, 2012; Hodson, 2014; Lamothe, 2014). In spite of the large number of existing devices, there has historically been a lack of published quantitative data on the biomechanics and energetics of humans using robotic lower limb exoskeletons (Lajeunesse et al., 2016). More studies on human user performance that compare devices or controllers would accelerate the development of improved devices in the future (Ferris, 2009).

Many lower limb exoskeletons are designed to assist walking such that the user lowers their muscle activity but remains in control over the movement (Lenzi et al., 2013). This results in the user modifying their joint torque to make the sum of the robotic assisted torque and biological muscle torque to be similar in magnitude to the torque during unassisted walking (Lewis and Ferris, 2011). By reducing the net joint torque required by the muscles, it may be possible to reduce the amount of mechanical power that muscles must provide to help reduce the metabolic cost of walking (Farris et al., 2013). In situations where the metabolic cost of walking is high (such as carrying a heavy load or pathological conditions), exoskeletons have the potential to reduce the metabolic cost of walking to a sustainable level (Panizzolo et al., 2016).

An important aspect of a robotic exoskeleton is that it must function in coordination with the movement of the body (Cenciarini and Dollar, 2011). The human and exoskeleton need to work in parallel so that the exoskeleton’s movement ideally does not conflict with or restrict human movement (Cenciarini and Dollar, 2011). It seems simple to state, but it is difficult in practice. The exoskeleton should be designed with the individual’s walking strategy in mind to adequately support and assist the body (Lenzi et al., 2013). In addition, the joint centers of the body and the exoskeleton should be aligned to prevent relative motion between the exoskeleton and the user (Cenciarini and Dollar, 2011).

Controller studies on testbed ankle exoskeleton systems (Ferris et al., 2006; Sawicki and Ferris, 2008; Malcolm et al., 2013; Farris et al., 2014b; Galle et al., 2015; Jackson and Collins, 2015; Kim et al., 2015; Koller et al., 2015a; Sawicki and Khan, 2015; Takahashi et al., 2015) and multi-joint exoskeleton systems (Neuhaus et al., 2011; Van Kammen et al., 2014; Cestari et al., 2015; Wang et al., 2015; van Asseldonk and van der Kooij, 2016; Stroppa et al., 2017) have helped enable the development of highly engineered, autonomous ankle (Meijneke et al., 2014; Mooney et al., 2014; Collins et al., 2015; Mooney and Herr, 2016; van Dijk et al., 2017) and multi-joint exoskeletons (Zoss et al., 2006; Sasaki et al., 2013; Hartigan et al., 2015; Kozlowski et al., 2015; Raab et al., 2016; Grasmücke et al., 2017) with promising results. Similarly, with hip exoskeletons, there is a need for testbed system results (Lewis and Ferris, 2011; Lenzi et al., 2013; Young et al., 2017) to help inform the control of new autonomous systems (Giovacchini et al., 2014; Buesing et al., 2015; Seo et al., 2016; Karavas et al., 2017; Sugar et al., 2017). Ankle exoskeleton studies on testbed devices have shown that human users lower their muscle force with added ankle exoskeleton assistance to keep the total joint moment around the ankle consistent with unassisted walking (Lewis and Ferris, 2011). Autonomous ankle exoskeleton developers have used this principle to reduce the energetic cost of walking with robotic ankle assist devices (Mooney et al., 2014; van Dijk et al., 2017). The ankle produces a large amount of the mechanical power during walking, but studies indicate that the hip joint contributes as much or more mechanical power (Sawicki et al., 2009; Farris and Sawicki, 2012; Meijneke et al., 2014). In the limited studies on hip exoskeletons, human users tend to reduce their own muscle joint torque and allow the robotic torque to substitute (Ding et al., 2017), as seen with ankle exoskeletons (Lewis and Ferris, 2011). In addition, there appears to be a trade-off between hip and ankle mechanical power in that both joints can compensate for each other during push-off (Lewis and Ferris, 2008; Lenzi et al., 2013; Koller et al., 2015a). Further testing to optimize hip exoskeleton controllers is needed to empower autonomous hip exoskeleton systems.

A common goal of human augmentation exoskeletons is to reduce the metabolic cost of walking (Ronsse et al., 2011; Mooney et al., 2014; Ding et al., 2016a; Ruiz Garate et al., 2016; Seo et al., 2016). Recent advances in the exoskeleton field have successfully reduced metabolic cost of walking in an exoskeleton below the metabolic cost of humans walking without an exoskeleton both with ankle exoskeletons (Collins et al., 2015; Mooney and Herr, 2016) and hip exoskeletons (Asbeck et al., 2015; Seo et al., 2016). The performance of these exoskeletons is a combination of both the hardware and control system. A wide variety of control systems have been proposed (Aguirre-Ollinger, 2013; Jang et al., 2015; Koller et al., 2015b; Oh et al., 2015; Takahashi et al., 2015; Wu et al., 2015; Yan et al., 2015; Ao et al., 2016; Chen et al., 2016; Ding et al., 2016b; Zhang et al., 2016) but rarely are direct comparisons made. For interested readers, recent reviews of the exoskeleton control literature are available that discuss controllers in detail (Tucker et al., 2015; Yan et al., 2015; Young and Ferris, 2017). Our study helps to bridge this gap by directly comparing control strategies on the same hardware and analyzing changes in metabolic cost.

Commercial exoskeleton systems have primarily focused on using input from mechanical sensors to provide control signals, but biological sensors that gain insight into the nervous system may be useful for exoskeleton control. The dominant commercial approach for robotic lower limb exoskeletons is to use kinematic and/or kinetic sensors to generate a control signal for actuators based on a finite state machine. Another major control approach for robotic exoskeletons is to connect to the user’s nervous system either directly through neural control (Gancet et al., 2012) or indirectly through myoelectric control (Kinnaird and Ferris, 2009). Gaining a direct sense of user intent with neural or myoelectric control can be highly valuable for feedforward control for exoskeleton technology. The HAL exoskeleton is the only commercial lower limb exoskeleton that currently uses myoelectric control (Kawamoto et al., 2003; Wall et al., 2015). Myoelectric control may have advantages over control systems using purely mechanical sensors in that it produces more natural movement and/or faster recognition of the user intent. Research groups have explored myoelectric control for exoskeletons (Andreasen et al., 2005; Ferris et al., 2006; Fleischer et al., 2006; Ao et al., 2016; Chen et al., 2016) and neural control (Gancet et al., 2012; Kilicarslan et al., 2013; Kwak et al., 2014; Soekadar et al., 2015) in the lab. Our goal was to directly compare direct proportional myoelectric control on a hip exoskeleton to a controller that supplies a nominal hip joint torque profile.

Direct comparisons between different controllers are rare; typically, exoskeleton hardware and software are developed in parallel with one controller per hardware platform (Young and Ferris, 2017). Studies directly comparing controllers are useful to devices coming onto the market such as the Honda Walking Assist device, which is a hip exoskeleton for rehabilitation (Buesing et al., 2015). A previous study by our group compared myoelectric control to providing a time-based plantarflexor burst at push-off for an ankle exoskeleton (Cain et al., 2007). The study found that myoelectric control provided smoother biomechanics and allowed subjects to reduce their muscle activation levels better than with the foot switch-based controller. The current study investigates a state machine-based controller that was designed to provide a biological hip torque profile during stance phase based on Winter’s biomechanical data from humans (Winter et al., 1996), which is a common strategy in the field (Ding et al., 2016a; Seo et al., 2016). We also designed a proportional myoelectric controller that bilaterally measures hip flexion and hip extension electromyography (EMG) signals to control the exoskeleton. Proportional EMG controllers have the capabilities of responding directly to the muscle commands of the user to provide a better response to dynamic changes in movement (Gordon et al., 2013; Grazi et al., 2015). In addition, EMG precedes force generation (Cavanagh and Komi, 1979) and thus may be a useful signal for exoskeleton control. Our previous experiments have successfully used proportional EMG control with ankle exoskeletons (Cain et al., 2007; Kinnaird and Ferris, 2009). We expected both controllers to reduce the metabolic cost of walking compared to unpowered walking. We hypothesized that the myoelectric control would reduce the metabolic cost of walking more than the biological torque control. We based this hypothesis on our previous study showing myoelectric control reduced muscle activation more than a kinematic state machine controller (Cain et al., 2007). We expected that the myoelectric control would feel more natural than state machine control to the users given its imitation of normal physiological function.

## Materials and Methods

### Device Design

**Figure 1:**
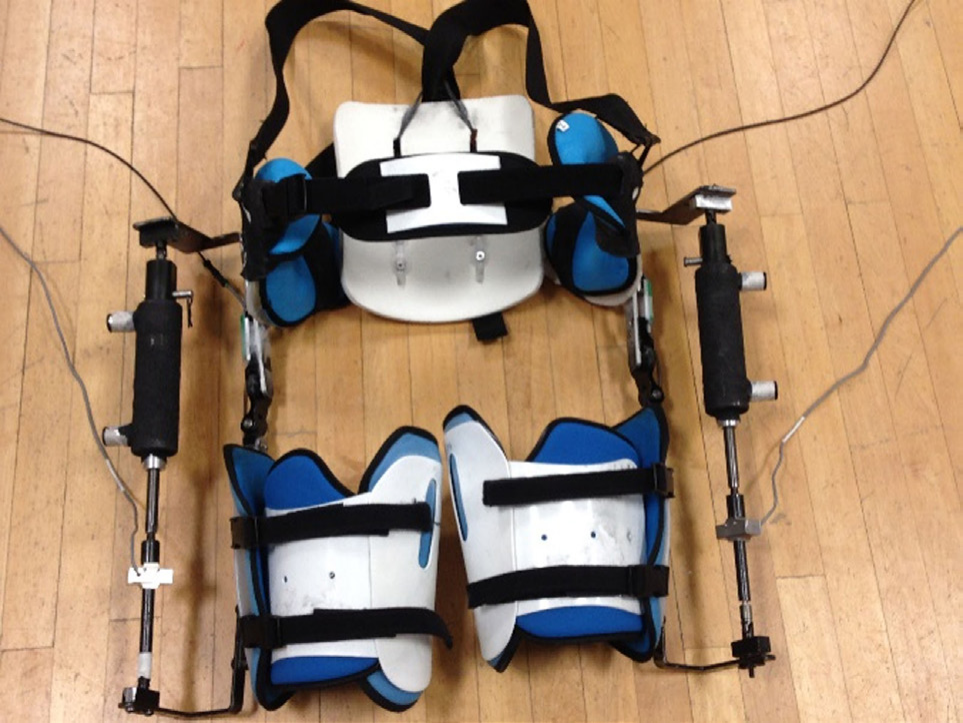
Pneumatic hip exoskeleton. The device included a waist unit that attached to the pelvis, padded thigh cuffs that were adjustable to the size of the user’s upper leg, a set of shoulder straps, a two degree of freedom hip joint, and pneumatic actuators to apply torque around the hip joint. Total weight of the hip exoskeleton was 6.8 kg.

We designed a hip exoskeleton device to apply torques around the user’s hip joint (Figure 1), with a total weight of 6.8 kg. The design was a modification of a previous exoskeleton frame from our lab (Lewis and Ferris, 2011). Bidirectional piston cylinder pneumatic actuators (BIMBA, University Park, IL, USA) on each leg provided hip flexion and extension assistance and were tethered to an external air tank at 620.5 kPa (90 PSI). Load cells (Omega) in line with the actuators measured the exoskeleton output force. The exoskeleton frame included a waist and thigh section with adjustable straps that secured the exoskeleton to the user. The exoskeleton actuated in the sagittal plane with a maximum torque capacity of 21 N m. The frontal plane had a passive joint to allow full range of motion. We attached electrogoniometers (Biometrics Ltd.) across the hip joint on the exoskeleton to measure the hip joint angle of the device.

### Controller Design

We tested two different control strategies in this experiment using a real-time control system (Control Desk, dSpace Inc.) on the exoskeleton hardware described above (see Device Design). The outputs from the controllers were signals to four pneumatic cylinders that corresponded to bilateral hip flexion and extension.

#### State Machine Controller

**Figure 2:**
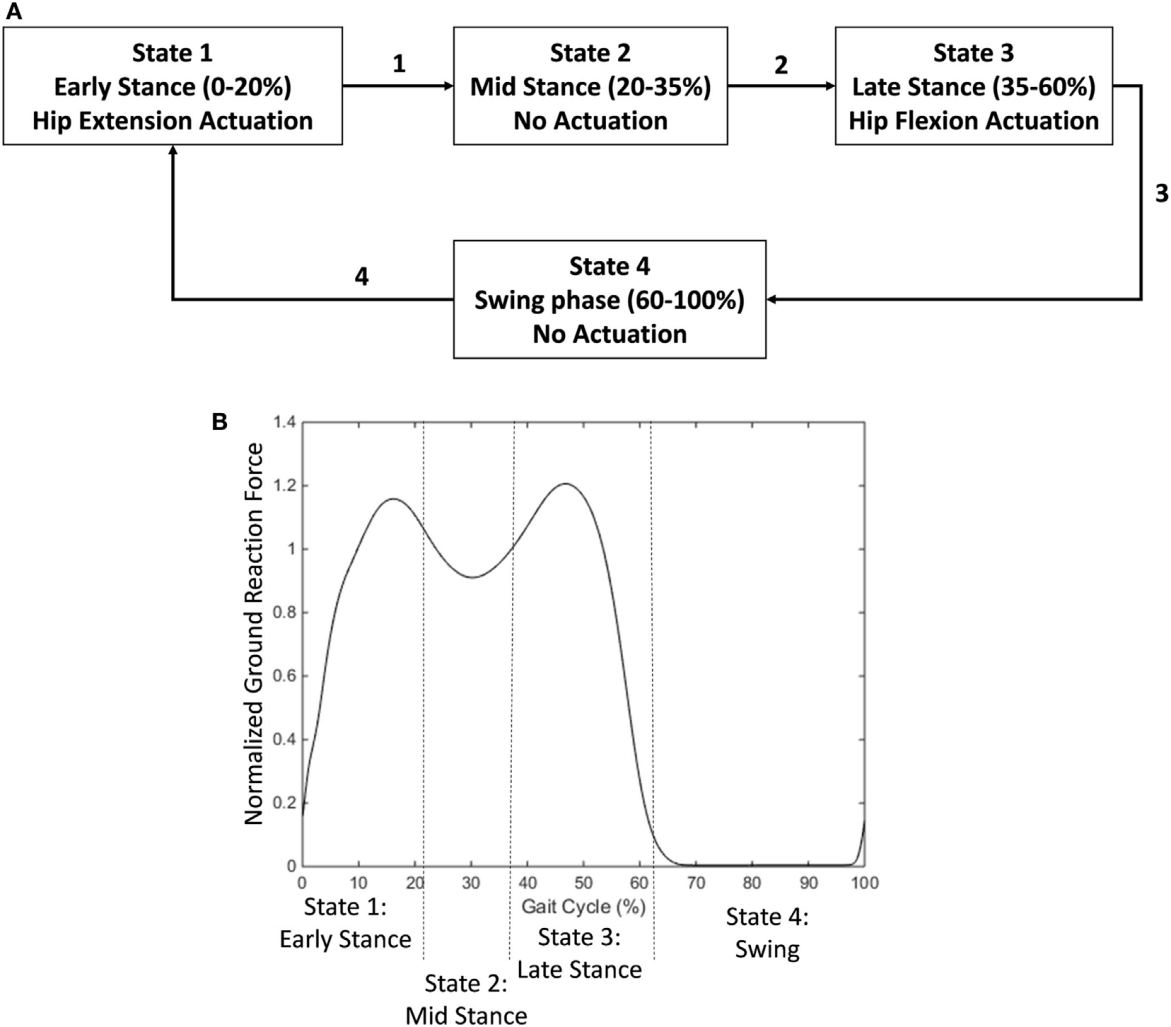
Diagram of state machine used to provide a biological hip torque profile. **(A)** Early stance phase (detected at the heel contact event) activated a hip extension torque. A transition to mid stance occurred at approximately 20% of the gait cycle. Mid stance was unpowered. A transition to late stance occurred at approximately 35% of the gait cycle. Late stance activated a hip flexion torque. Toe off triggered a transition to swing phase which was not actuated. **(B)** States relative to the ground reaction force profile (normalized to body weight). A threshold trigger switched the states between stance and swing phase (15% of body weight). Mid stance was triggered based on a decrease from the first peak in the ground reaction force profile. Late stance was triggered based on an increase in the ground reaction force profile from the trough.

The first control strategy was a state machine controller that provided an exoskeleton torque profile similar to a standard biological hip torque profile. The hip joint torque during human walking is characterized by two key features from biomechanical data (Neumann, 2010). The first is an extension torque peaking at ~10% of the gait cycle. The second is a flexion torque peaking at ~50% of the gait cycle. The state machine controller used mechanical signals from the hip angle and ground reaction force to calculate gait phase and apply an appropriate torque profile. It had four states to control joint torque levels (Figure 2A).

##### State 1—Early Stance

The first state occurred during early stance phase from approximately 0–20% of the gait cycle. During this phase, the exoskeleton provided active hip extension assistance with a maximum supply signal at full hip flexion and linearly decreased to 0 as the hip extended to the neutral position (Eq. 1).

(1) $$\begin{array}{l} \text{Supply Pressure }=\frac{\text{Current Hip Angle}}{\text{Maximum Hip Flexion Angle }}\times \text{ Maximum Supply Pressure} \end{array}$$

The maximum hip flexion angle occurs at heel contact. This parameter varies slightly per subject but was approximately 30°. It is set once per subject at the beginning and does not require further tuning. The current hip angle is read by a goniometer. The output is 0 if the hip angle is negative (in extension). The maximum supply pressure that our air tank was able to afford is 90 psi. The pneumatic actuator is powered at 100% at the beginning of the phase and linearly decreases to 0 with hip angle as the hip extends during stance. This relationship is very similar to the hip angle/joint torque relationship from the literature.

##### State 2—Mid Stance

The second state occurred during the mid-stance phase from approximately 20–35% of the gait cycle. During this phase, the exoskeleton was unpowered.

##### State 3—Late Stance

During the third state, from approximately 35–60% of the gait cycle, the exoskeleton provided active hip flexion assistance with a supply signal linearly related to the subject’s weight on the stance leg. As the subject removed weight from the leg, the supply signal linearly decreased to 0 based on the force measurement (Eq. 2).

(2) $$\begin{array}{l} \text{Supply Pressure }=\frac{\text{Current Vertical Force}}{\text{Maximum Vertical Force }}\times \text{ Maximum Supply Pressure} \end{array}$$

The output hip flexion scales linearly with vertical weight. The maximum vertical force was set at the beginning of the experiment and was different based on the subject’s weight (approximately 1.2 times body weight). This force corresponds to the second peak in the vertical ground reaction force profile. The supply pressure scales linearly with applied weight. Thus, the state has a large flexion torque applied during the beginning with the rise of the second peak of vertical ground reaction force and drops off as the person takes weight off the leg and transitions into swing.

##### State 4—Swing

The exoskeleton was unpowered in the fourth state (swing phase).

The transitions in the state machine were determined by the user’s ground reaction force profile (Figure 2B). The state machine controller determined stance and swing phases based on a threshold value (15% of body weight). The transition between early stance and mid stance was determined based on a 5% drop in the vertical ground reaction force signal from the first peak of the profile. The transition between mid stance and late stance was determined based on a 5% increase in the ground reaction force signal from the middle trough of the profile.

#### EMG Controller

The second type of controller tested was a proportional myoelectric controller. EMG electrodes on the gluteus maximus and rectus femoris provided muscle activation signals for the controller. The controller used gluteus maximus EMG to activate hip extension assistance while rectus femoris EMG activated hip flexion assistance. The rectus femoris is not an ideal muscle for hip flexion assistance because it is biarticular, but other hip flexion muscles, such as the iliopsoas and the sartorius, are often hard to record with surface electromyography. To deal with co-contraction (where both muscles were active), we used an override such that the extensor EMG signals were used in late swing and early stance, while the flexor EMG signals were used in late stance and early swing. EMG signals were first smoothed by high pass filtering at 40 Hz, rectifying and then low pass filtering at 6 Hz. For each of the four muscle locations (left and right rectus femoris and left and right gluteus maximus), a threshold and a gain were set.

$$\begin{array}{c} \text{Supply Pressure}=\text{EMG gain}\times (\text{Current Processed EMG value}-\text{ EMG threshold}) \end{array}$$

**Figure 3:**
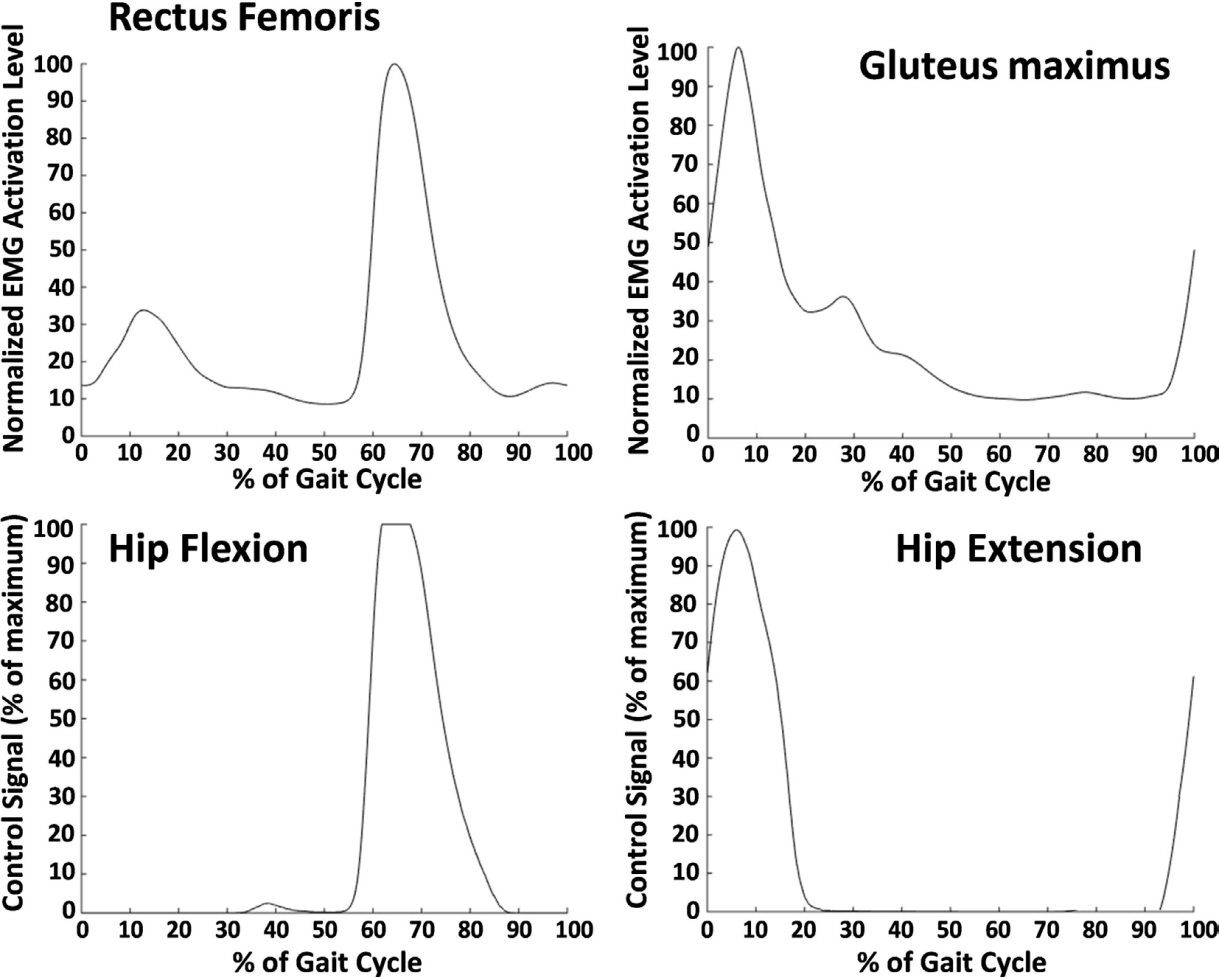
Average electromyography (EMG) signals (top row) and average control signals (bottom row) from one subject. These data were averaged over 30 min of walking. The top left shows the smoothed EMG signal from the rectus femoris (normalized to the maximum value during the gait cycle), and the top right shows the smoothed EMG signal from the gluteus maximus. The rectus femoris signal was used to determine the control signal for hip flexion, and the gluteus maximus was used for hip extension. The burst in rectus femoris activity in early stance was ignored by the controller. A gain and a threshold were manually set for each muscle and each subject to ensure that the peak muscle activity corresponded with a maximum supply signal output. The resulting control signal for hip flexion is shown in the bottom left and for hip extension in the bottom right.

The threshold, which was set while the subject stood quietly, was set to ensure that the baseline activity when the muscle was not active did not trigger assistance. The gain was set to allow the peak of the muscle activity during each stride to reach a 100% supply pressure signal. This was done by having the subject walk and then adjusting the gain such that the peak of the EMG signal during a stride aligned with maximum output supply signal for the pneumatic actuators. An example of rectus femoris activity converted to the air supply signal is shown in Figure 3. The gluteus maximus has only one peak that occurs in early stance that was translated to a hip extension control signal. The hip flexion control signal was only active during the second peak—associated with hip flexion—of the rectus femoris activity. The first peak—associated with knee extension—was overridden by the extension signal.

### Experimental Protocol

**Figure 4:**
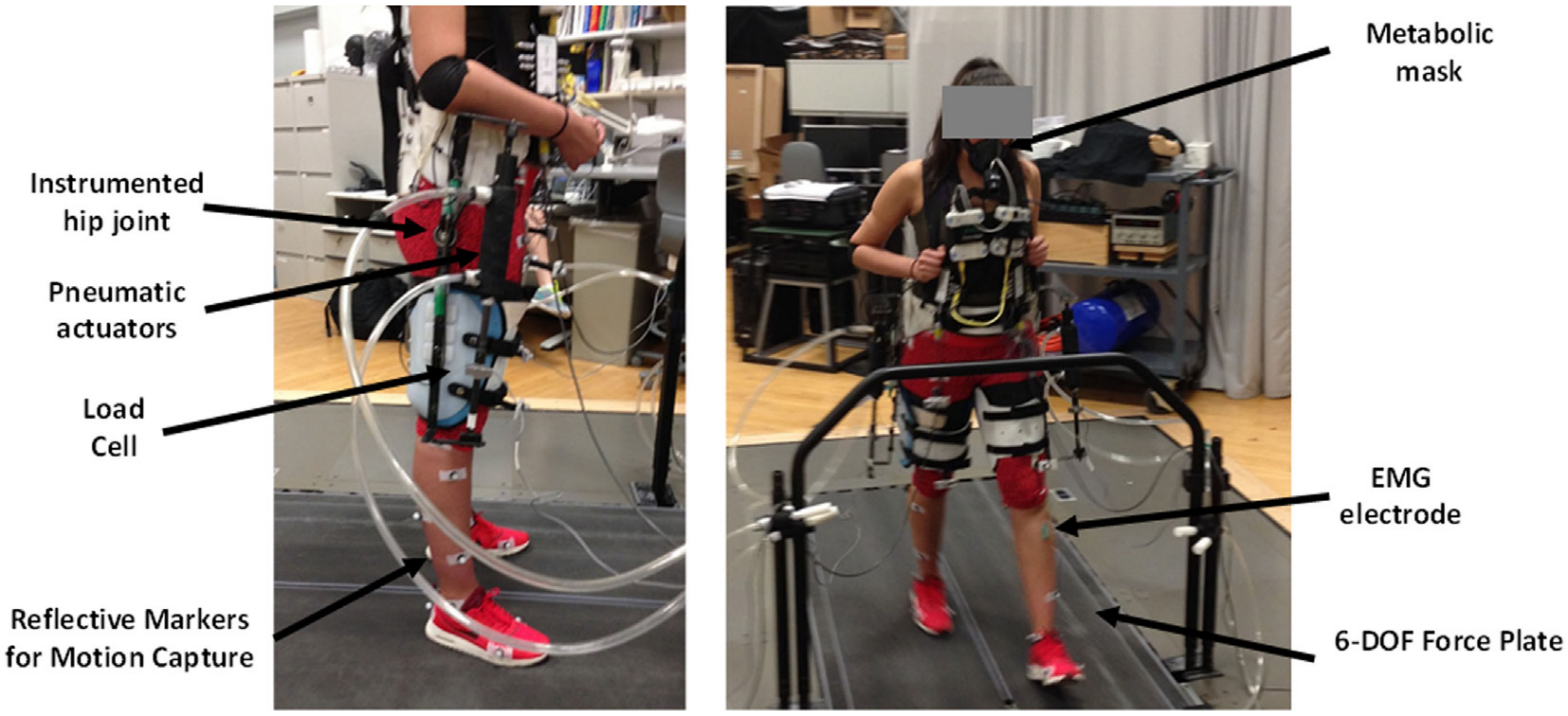
Experimental setup. Electrogoniometers recorded the exoskeleton hip joint motion. Pneumatic actuators produced flexion and extension forces recorded by load cells in series with the actuators. Motion capture recorded motion of the exoskeleton user. We recorded oxygen consumption and carbon dioxide production with a portable metabolic measurement system. We used electromyography (EMG) electrodes on the lower limbs to record muscle activation patterns. The treadmill had 6-DOF force plates to record ground reaction forces.

Ten able-bodied subjects [five males/five females, weight: 67.6 kg (8.8 SD)] gave written informed consent to participate and have their data published for the following experiment that had been approved by the University of Michigan Institutional Review Board. The experimenters located the following nine EMG electrode locations on the lower limbs of each subject: (1) right rectus femoris, (2) right gluteus maximus, (3) left rectus femoris, (4) left gluteus maximus, (5) left sartorius, (6) left medial hamstring, (7) left medial gastrocnemius, (8) left soleus, and (9) left tibialis anterior. The rectus femoris and gluteus maximus locations were control sites while the other six EMG locations were solely for the purpose of understanding changes in lower limb muscle activity during the experiment. The experimenters placed reflective markers along the lower limb for inverse dynamics calculations based on the Cleveland Clinic convention [Heisenberg, (date unknown)]. These marker locations included the feet, shank, knee, thigh, hip, and pelvis. We fit subjects to the exoskeleton using adjustable shoulder, waist, and thigh straps. The complete experimental setup with the different measurements that were made is shown in Figure 4.

Experimental testing was done on a single day. Subjects stood in the exoskeleton for 3 min while we measured oxygen consumption and carbon dioxide production using a metabolic analysis system (Oxycon Mobile, CareFusion). The subjects walked at 1.0 m/s to simulate a medium to low walking speed on a split belt-instrumented treadmill (Bertec) to measure their ground reaction forces. The order of the two powered conditions was randomized such that five subjects performed the state machine controller first and five subjects performed the EMG controller first. During the first 5 min of walking, subjects acclimated to walking with an exoskeleton as the experimenter slowly increased the amount of power supplied to maximum levels. Subject specific tuning parameters were set during this phase. For EMG control, the subject tuning parameters were the gains and thresholds for each of the four control muscles. For state machine controller, only the maximum hip flexion angle, the neutral hip flexion angle (measured during standing), and the peak weight were used as subject specific parameters for the controller. After the initial 5-min adjustment period, subjects walked for 30 min in each of the powered conditions while we measured their metabolic expenditure rate. In between the two powered conditions, subjects walked for 10 min with the unpowered exoskeleton while we measured their metabolic expenditure rate to have a baseline for comparison. Movie demonstrations of both controllers are available in Video S1 in Supplementary Material.

At the end of the experiment, subjects were verbally asked the following series of questions, and their responses were recorded. (1) Which controller (the first one or the second one) did you prefer to walk with? (2) Did you find the first controller to be overall helpful or not helpful for aiding your walking movements? Use the unpowered condition as reference. (3) Did you find the second controller to be overall helpful or not helpful for aiding your walking movements? (4) What were your general impressions of both controllers and what did you like and dislike about each of them? The last question (#4) was a more open ended question that allowed subjects to describe the controllers in their own words and provide general feedback to us about the control strategies.

### Data Analysis

We normalized data to the gait cycle by segmenting at heel strike. For each subject, data were averaged across all the walking strides of each trial. We calculated the across subject averages and SDs using the average value at each time point during the gait cycle for each subject. All data collected are from users using the exoskeleton device described in Section “Device Design.”

#### Controller Analysis

We calculated the average hip flexion and hip extension control signal across subjects to show the typical controller behavior during the experiment. We also calculated the torques generated by the controller on the exoskeleton by multiplying the moment arm measured on the exoskeleton with the force values obtained by the load cell. We determined the kinematics of the exoskeleton (hip angle in sagittal plane) using electrogoniometers instrumented on the hip exoskeleton. Based on the time derivative of the hip angle profile and torque profile, we also calculated exoskeleton power.

We evaluated the accuracy of the state machine transitions by calculating at which percent of the gait cycle a state transition occurred. The early stance to mid-stance transition was intended to occur at approximately 20% of the gait cycle. We defined an error if the transition occurred before 10% or after 30% of the gait cycle. The mid stance to late stance transition was intended to occur at 35% of the gait cycle. We defined an error if the transition occurred before 25% or after 45% of the gait cycle. We evaluated errors to check if the controller was deviating from the intended action.

#### Metabolic Analysis

We calculated the metabolic cost of walking in Watts using standard equations based on oxygen consumption and carbon dioxide production (Brockway, 1987). We subtracted the standing metabolic rate from the walking metabolic rate to calculate the net metabolic cost of walking in each condition. Metabolic data from the last 6 min of each walking trial were used to calculate each subjects’ metabolic energy expenditure during state machine control, EMG control, and unpowered walking.

#### EMG Analysis

The EMG system had a hardware filter between 15 and 450 Hz, and we digitized the signal at 1,000 Hz. Our real-time controller processed the EMG signals by high-pass filtering at 40 Hz, full wave rectification, and low-pass filtering at 6 Hz to smooth each signal. In postprocessing, we took the linear envelope of the EMG signals from each stride and averaged them together for each condition. We normalized the signals to the peak of the unpowered condition’s linear envelope and averaged across subjects. We compared peak normalized EMG activity during the gait cycle across conditions. EMG curves were averaged from strides from the last 6 min of walking in each condition.

#### Biomechanical Analysis

We recorded motion capture data in Vicon (100 Hz) using a 10 camera system. We performed marker tracking in Vicon and biomechanical analysis using Visual 3D (C-Motion). In Visual 3D, motion capture data were low pass filtered at 6 Hz before joint angles were calculated for the ankle, knee, and hip. Visual 3D calculated joint moments and powers at the ankle, knee, and hip with inverse dynamic models using both marker and force plate information. We excluded 2 of the 10 subjects due to the exoskeleton blocking their markers during the walking trials such that biomechanical analysis was impossible. Two minutes of biomechanics were recording during the last 6 min of walking, which were averaged and used to produce the graphs. We used the peak internal joint moments and powers during walking to compare results across conditions.

#### Statistical Analysis

We used repeated measures one-way ANOVA (Minitab 17) to test for significant differences in metabolic, EMG, and biomechanics data across the three different conditions (state machine control, EMG control, and unpowered walking). *Post hoc* Bonferroni tests (α = 0.05) corrected for multiple comparisons and differentiated conditions that were statistically different from each other. In addition, we performed a repeated measures two-way ANOVA to test for significant differences in metabolics over the 30 min time interval (in 5 min increments) where one factor was controller and the other time point.

## Results

### Metabolic Results

**Figure 5:**
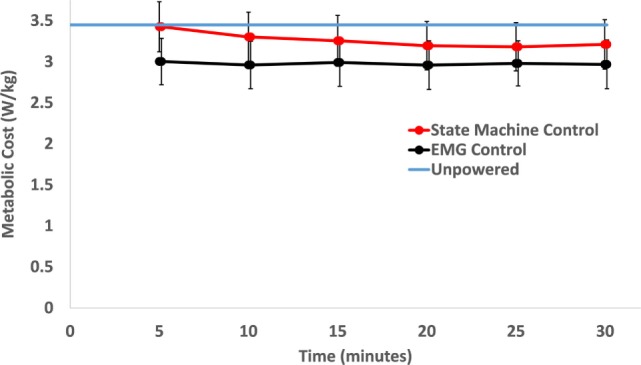
Metabolic cost of walking over time. Each time point includes the average of the previous 5 min of walking. Over the time course, only the state machine control condition had a significant decrease in metabolic cost between the first time point (at 5 min) and the last three time points (*p* = 0.002 between 5 and 20 min, *p* = 0.001 between 5 and 25 min, and *p* = 0.006 between 5 and 30 min). Within the condition, electromyography (EMG) control had no significant change over the course of the 30 min trial. The unpowered condition’s metabolic rate is shown as the blue line only for reference. Data were averaged across 10 subjects and error bars show ±1 SEM.

**Figure 6:**
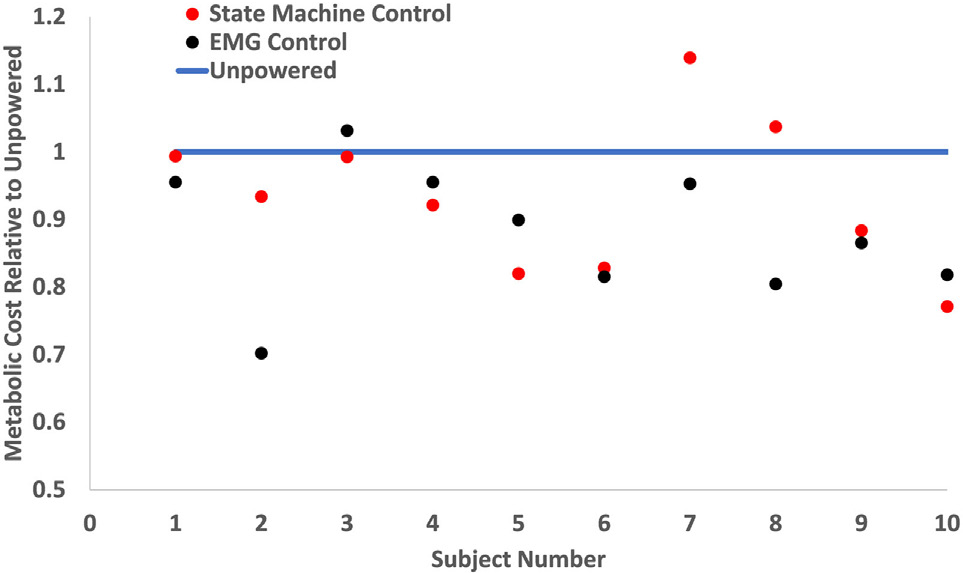
Metabolic cost of walking on a per subject basis. The metabolic costs were normalized to the unpowered condition’s metabolic rate (blue line). Red dots show the metabolic cost of state machine control, and black dots show the metabolic cost of electromyography (EMG) control. This graph shows each individual subject’s metabolic performance and indicates that the powered conditions consistently outperformed the unpowered condition. However, while EMG control had lower metabolic cost on the majority of subjects, this was not always the case for each subject.

The powered control conditions had lower metabolic cost than the unpowered condition, and the EMG control had lower metabolic cost than the biological torque control across subjects. The average metabolic cost of walking across the 10 subjects during the last 6 min of each condition was 3.42 (mean) ± 0.16 (SEM) W/kg for unpowered walking, 3.18 ± 0.17 W/kg for state machine control, and 2.96 ± 0.18 W/kg for EMG control. EMG control significantly (*p* = 0.005) reduced metabolic cost by 13% compared to the unpowered condition, while the state machine control reduced metabolic cost by 7% compared to the unpowered condition (not statistically significant, *p* = 0.261). The metabolic cost of walking over the 30 min trials for the state machine control found a significant reduction (*p* < 0.01) from the first time point (at 5 min) compared to later time points (see Figure 5), but there were no significant differences from the 10 min time point until the end of the 30 min duration. Powered controllers usually had lower metabolic cost than the unpowered condition across subjects (Figure 6).

### Biomechanical Results

**Figure 7:**
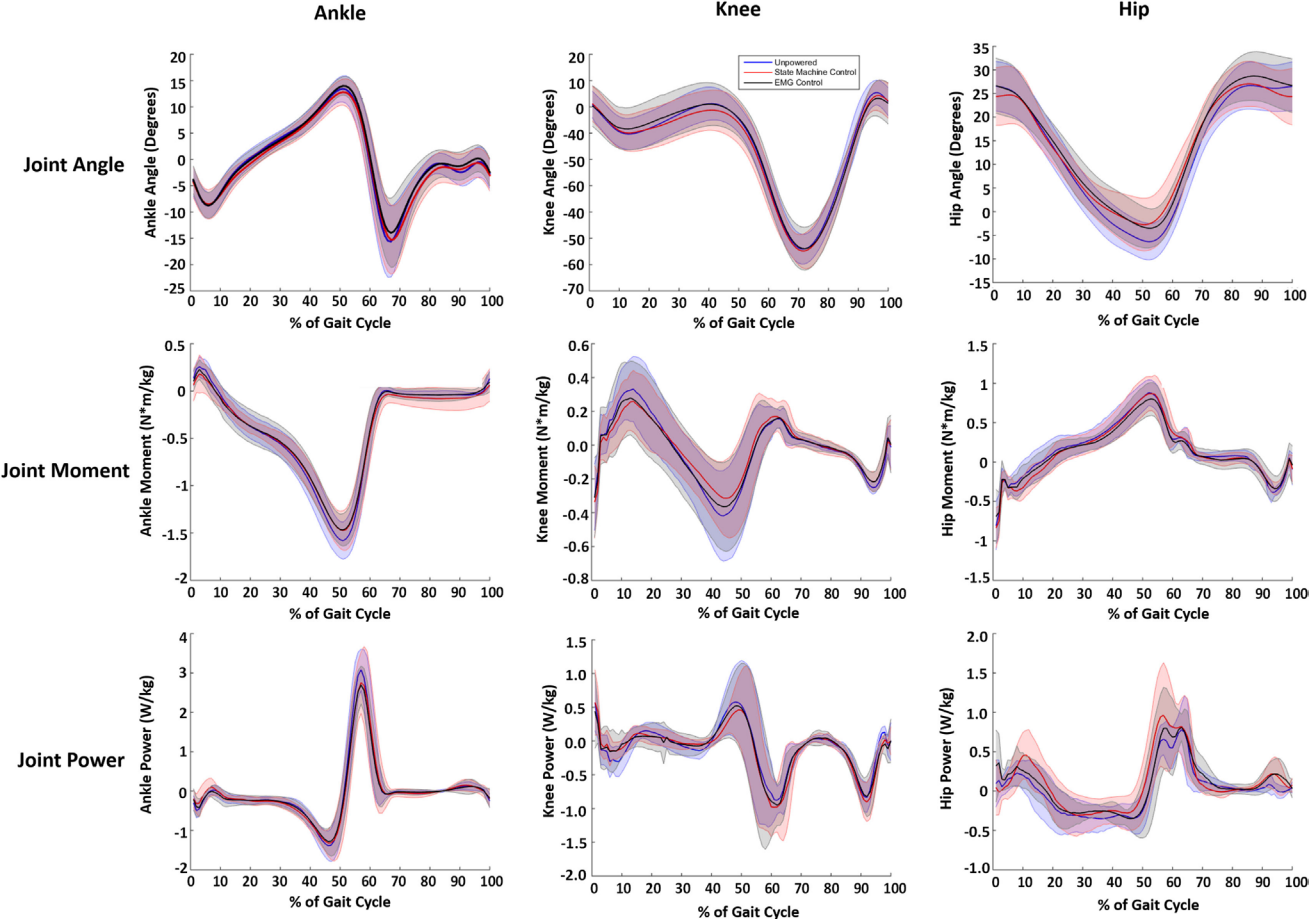
Joint kinematic and kinetics across the three walking conditions. Blue lines correspond to unpowered, red lines to state machine control, and black lines to electromyography (EMG) controls. It is important to note that the joint torques and powers presented are a combination of exoskeleton and human joint torque and power. The first column corresponds to the ankle, the second to the knee, and the third to the hip. The joint angles are in the first row. The second row shows the internal joint moments. The third row is the internal joint powers. Data were normalized to the walking cycle such that 0% corresponds to heel strike. Data were averaged across eight subjects, and shaded regions represent ±1 SD.

While a few trends were observed in subjects’ biomechanics, their walking profiles at the hip, knee, and ankle were largely similar across conditions (Figure 7). We note that the changes in biomechanics were small relative to the intersubject SDs and differences were not statistically significant, which indicated the exoskeleton torque largely replaced biological hip torque. The internal joint moments and powers at the ankle had slightly lower peaks (~10%) in the powered conditions compared to the unpowered condition (see Table 1). The internal joint moments at the knee were also reduced during stance phase for the powered conditions compared to unpowered (Table 1). The internal joint moments at the hip were lower in powered conditions compared to unpowered, but internal joint powers were larger with relatively high intersubject variability (Table 1). The kinematics at the ankle and knee were largely similar across subjects. However, a major kinematic difference occurred at the hip with the powered controllers. Both powered controllers lowered the amount of excursion into hip extension at ~50% of the gait cycle from ~7° to ~3° compared to unpowered walking.

**Table 1:** Average peak joint moment (N m/kg) and associated peak joint positive power (W/kg) for the ankle, knee, and hip for each condition.

Joint	Biomechanical variable	Unpowered	State machine control	Electromyography control
Ankle	Peak plantarflexive moment	1.59	1.46	1.46
	Peak positive power	3.05	2.72	2.65

Knee	Peak flexion moment	0.42	0.31	0.36
	Peak positive power	0.58	0.45	0.52

Hip	Peak flexion moment	0.86	0.85	0.79
	Peak positive power	0.77	0.93	0.80

### EMG Results

**Figure 8:**
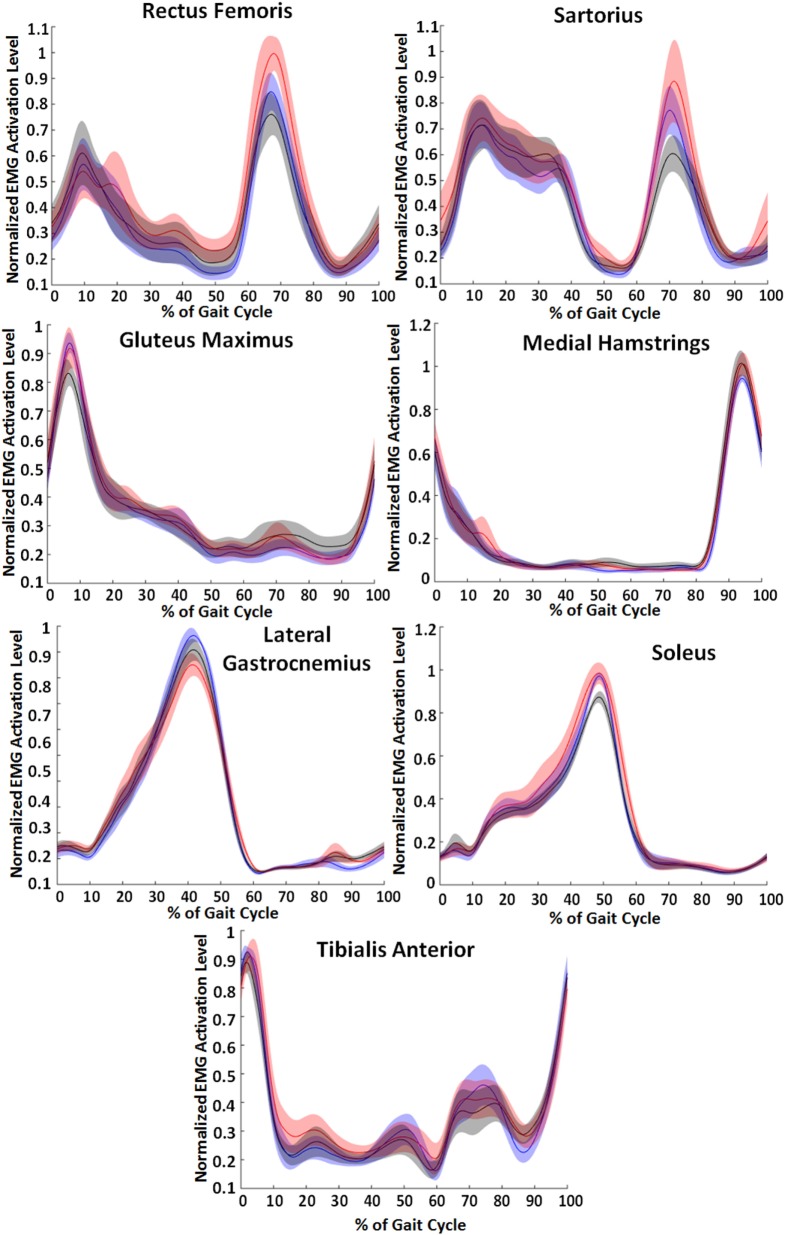
Lower limb electromyography (EMG) activity across the three walking conditions. Blue lines correspond to unpowered, red lines to state machine control, and black lines to EMG controls. Data were smoothed for each muscle and normalized to the maximum of each of the individual subject’s unpowered walking condition. Data across the 10 subjects were averaged, and shaded regions represent ±1 SD. Data were normalized to the walking cycle such that 0% corresponds to heel strike.

Muscle activation patterns generally showed the lowest values for the EMG control condition. Figure 8 shows the normalized EMG patterns across the three conditions—unpowered, state machine control, and EMG control. Rectus femoris and sartorius muscle activity had differences in the peak EMG signal that occurred between 65 and 70% of the gait cycle, a peak corresponding to hip flexion activity. At this peak, the EMG condition tended to decrease activity while the state machine condition tended to increase activity. The peak of gluteus maximus EMG activity occurred just after heel strike and slightly lower in the powered conditions than unpowered. Hamstring activity peaked at 95% of the gait cycle and had a slight increase in both the powered conditions compared to unpowered. The peak in gastrocnemius EMG activity occurred at 40% of the gait cycle and was 6% less in the EMG condition and 12% less in the state machine condition (*p* = 0.008) compared to unpowered. The peak in soleus EMG activity occurred at 50% of the gait cycle and was 8% less in the EMG condition (*p* = 0.028) compared to unpowered and the state machine controller which were the same. The tibialis anterior had two peaks, the first occurred between 65 and 80% of the gait cycle and had a 14% smaller peak in the EMG condition (*p* = 0.02) and 10% smaller peak in the state machine condition across subjects compared to unpowered. The second occurred immediately following heel contact and had only slightly decreased peaks in the powered conditions compared to unpowered.

### Controller Results

**Figure 9:**
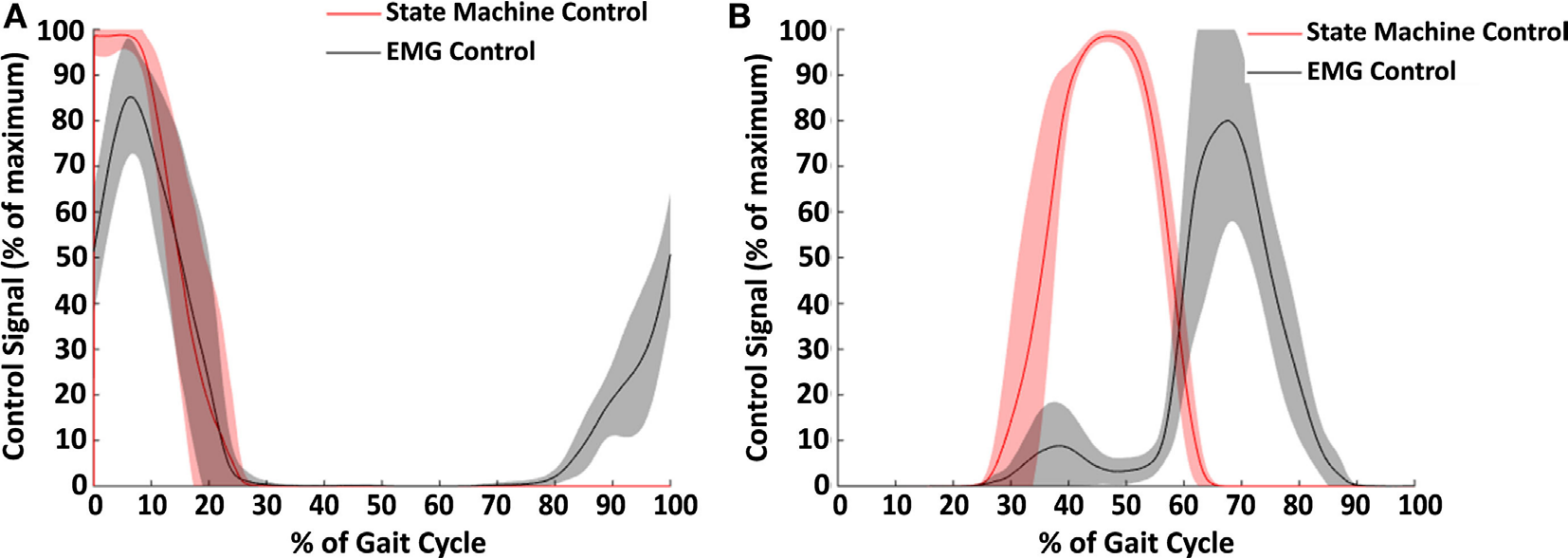
Control signals across subjects for hip extension **(A)** and hip flexion **(B)**. The average control signal for the state machine control condition is shown in red and for the electromyography (EMG) control condition in black. Shaded regions show ±1 SD. This figure demonstrates the difference in strategy between the two controller conditions. Notably, the EMG control has an earlier onset in hip extension, and the state machine controller has an earlier onset in hip flexion.

The two types of controllers tested in this study, EMG control and state machine control, generated different signals for both hip extension (Figure 9A) and flexion (Figure 9B). The state machine generated a maximum supply signal at heel contact which decreased linearly based on exoskeleton hip angle until turning off at 20–25% of the gait cycle. In contrast, the EMG control began generating a signal prior to heel contact based on the gluteus maximus EMG activity. The stance phase portion of the extension control signal was very similar with EMG control and state machine control. For hip flexion control, the state machine peaked at approximately 45% of the gait cycle, while the EMG peaked between 65 and 70% of the gait cycle. Thus, the timing of hip flexion assistance was substantially different between the two controllers.

**Figure 10:**
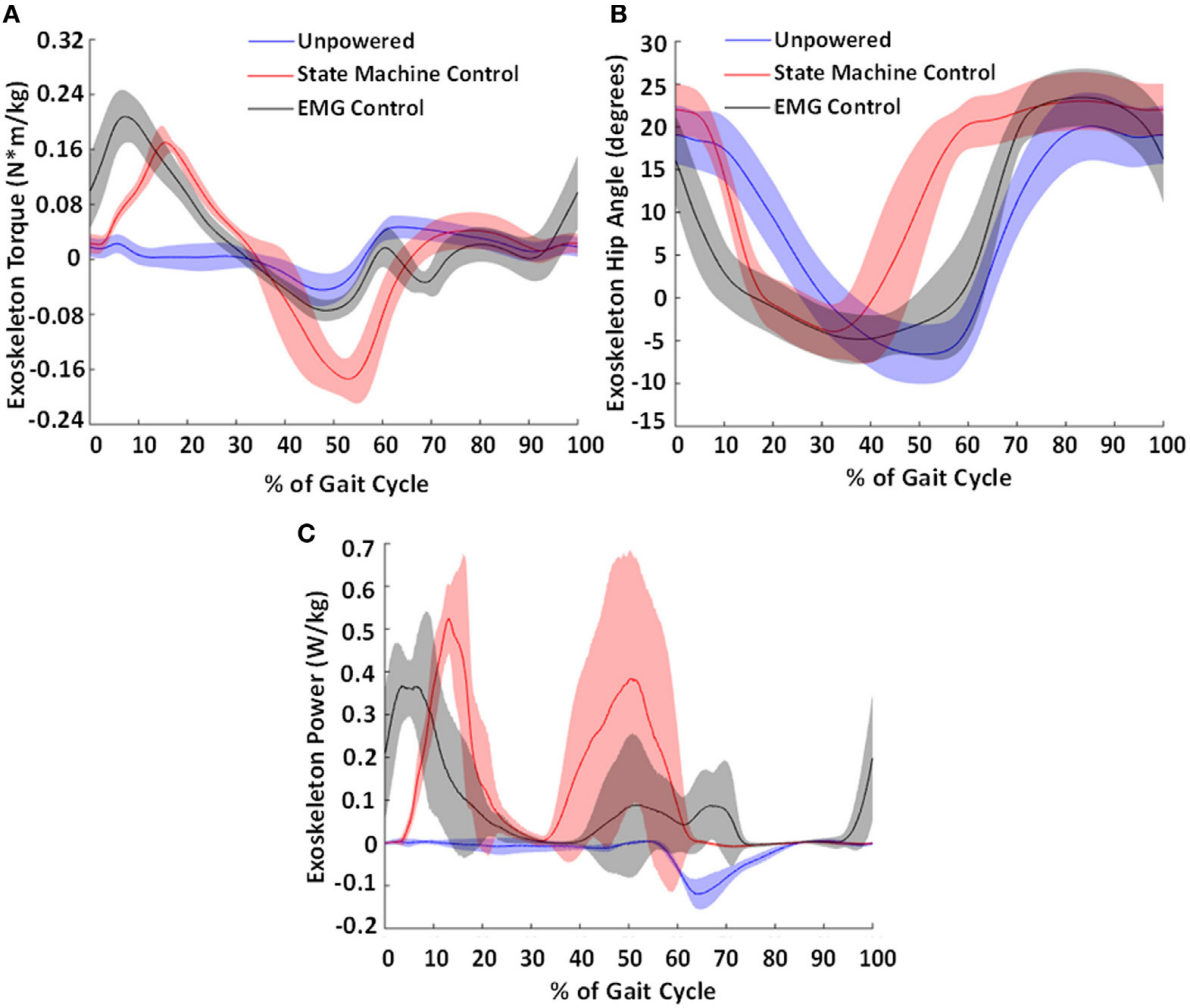
Torque produced by the exoskeleton **(A)**, exoskeleton hip angle **(B)**, and calculated exoskeleton power profile **(C)**. The average torque and power generated by the exoskeleton (normalized to body weight) and exoskeleton hip angle across the subjects for the state machine control condition is shown in red and for the electromyography (EMG) control condition in black and the unpowered condition in blue. Shaded regions show ±1 SD. This figure demonstrates the differences in output torques and powers produced by the different controllers. These resulted in different hip exoskeleton angle profiles. Notably, the earlier onsets in control signal (shown in Figure 9) resulted in an earlier torque and power generation in the exoskeleton.

The differences in controllers resulted in different profiles of torque and power produced by the exoskeleton onto the user (Figure 10A). The state machine effectively replicated an exoskeleton torque profile during stance phase similar to that of a biological torque profile, at approximately a third the magnitude of biological levels (Neumann, 2010). The EMG controller had a larger extension torque that occurred earlier in the gait cycle. This was due to the ramp up of the gluteus maximus activity during late swing. The EMG controller’s peak flexion assist torque occurred at 69% of the gait cycle but was much smaller in magnitude compared to the state machine’s peak torque that occurred at 53% of the gait cycle. This was partially because the hip joint velocity was low when the state machine began generating torque, allowing a larger peak magnitude. However, during the EMG assistance time point (at 69% of the gait cycle), the hip was actively moving in flexion which reduced the amount of torque the pneumatic actuators were able to provide to assist hip flexion.

The exoskeleton kinematics (Figure 10B) indicate that the exoskeleton motion followed the control signals as expected. During hip extension, the exoskeleton moved faster with EMG control compared to state machine control due to the earlier onset of hip extension assistance applied with EMG control. Similarly, during hip flexion, the exoskeleton moved faster into flexion with state machine control when compared with EMG control due to the earlier onset of hip flexion assistance in state machine control. Compared to the human hip joint kinematics (Figure 7), it is clear that there is movement of the hip exoskeleton relative to the person, especially in the powered conditions. This is likely because the applied torques press into the soft tissue of the thigh during actuation.

### Accuracy of the State Machine

Overall, the state machine was highly accurate for determining phase transitions with 100% accuracy between stance and swing states. Transitions out of early stance occurred too early in 0.32% (0.30 SEM) of steps and had the effect of turning off the hip extension control signal before it was intended, thus delivering a lower overall extension torque. Transitions out of early stance occurred too late in 0.19% (0.15 SEM) of steps and had the possible effect of delaying the turning on of the hip flexion control signal. Transitions out of mid stance occurred too early in 0.19% (0.10 SEM) of steps and had the effect of turning on the hip flexion control signal before it was intended, thus delivering a larger overall hip flexion torque. Transitions out of mid stance occurred too late in 0.04% (0.02 SEM) of steps and provided substantially less or no hip flexion torque. The average error rate of the state machine was 0.74% (0.50 SEM) across subjects.

### Subjective Subject Preferences

8 out of 10 of the subjects preferred the EMG control condition over the biological torque control condition. In general, based on the subjective feedback, most subjects liked that the biological torque control felt regular and predictable. The two subjects that preferred the biological torque control condition gave this as the reason. Other subjects said that although it was predictable, they also felt constrained to either a specific pace or movement pattern. Subjects tended to prefer the EMG condition and have a better overall experience due to it either feeling “smoother,” being more in tune to their movements, or that they could walk more easily in line with it. In general, subjects seemed to like the timing of the EMG controller better, but also were very aware that it was less predictable. This decreased predictability was favored by some subjects but cited as a disadvantage by others. All 10 subjects thought that both powered conditions were helping more than they were not helping. One subject summarized it by saying that they felt like the exoskeleton was very heavy in the unpowered condition, but this weight was not nearly as noticeable in the powered conditions (even though the overall exoskeleton weight was the same in all conditions). Interestingly, subject preference did not seem to correspond well to metabolic cost between the two powered controllers as only 4 out of 10 subjects preferred the control condition with a lower metabolic cost.

## Discussion

### Comparison of Control Strategies

The findings from this study suggest that there may be some advantages for proportional myoelectric control for controlling robotic exoskeletons compared to a standard biological torque-based controller. The EMG controller had a larger metabolic reduction (13% lower than unpowered) (*p* = 0.005) than the biological hip torque control (7% lower than unpowered). However, some subjects had lower metabolic cost with state machine control than with EMG control (Figure 6). There was substantial subject-to-subject variability in the metabolic results. A possible explanation for this variability was that subjects may have adopted different strategies depending on the controller. In addition, subjects’ ability to adapt to a given controller may have contributed to the variability. Some subjects felt they were sometimes fighting with the exoskeleton, but many were able to adapt to each controller and perceived they were being helped by the exoskeleton. We gave subjects a total of 35 min of walking in each control condition and metabolic cost stabilized long before the end of this walking period. However, it is possible that with additional training, especially incorporating targeted feedback (Huang et al., 2016), some subjects may have been able to have superior performance.

Previous literature has found that adding weight on subjects yields a linear increase in metabolic cost during walking (Taylor et al., 1980; Bastien et al., 2005). In our experiment, the exoskeleton increased the total weight of the users by 10% on average. Based on this inference and comparison to nominal values from the literature (Bastien et al., 2005), the biological hip torque controller did not reduce metabolic cost by enough to account for the weight penalty while the EMG controller reduced metabolic cost slightly below the 10% threshold. The differences between the two controllers were also evident in the average EMG activity across the seven lower limb muscles that we measured (Figure 8). The EMG controller had lower peak EMG activity in six of the seven muscles measured compared to the unpowered condition. In contrast, in biological hip torque control only two muscles—the medial gastrocnemius and the tibialis anterior—had lower peak activities relative to unpowered. In addition, hip flexion activity appeared to have increased in biological hip torque control relative to unpowered based on increases in both the rectus femoris and sartorius. This indicates that the hip flexion assistance provided by the biological hip torque control may have not been useful to the subjects and may have even caused increased use of hip flexors.

We observed small, but consistent changes in overall biomechanics based on the control condition. One of the largest changes was that the powered exoskeleton conditions did not allow full hip extension, which is difficult to prevent from a control perspective. Previous research has suggested that humans try to keep the net moment of the knee, ankle, and hip at a consistent level (Winter, 1980)—even though each individual joint varies considerably. The slight changes in ankle and knee kinetics is similar to related research at the ankle where plantarflexor torque delivered by the exoskeleton resulted in reduced torque profiles at both the ankle and hip (Koller et al., 2015b). These results correspond with previous hip exoskeleton research (Lewis and Ferris, 2011; Lenzi et al., 2013). Thus, the robotic hip torques provided by the hip exoskeleton may have slightly reduced the net moments and powers at the ankle and knee joint. However, the biomechanics results indicate that the powered exoskeleton device largely substituted robotic torque and power for biological torque and power at the hip joint, similar to prior related work with hip exoskeletons (Lewis and Ferris, 2011).

The biomechanical results showed that the control condition did not have a large effect on the combined human and exoskeleton joint torques and powers, which are in agreement with prior related work (see Introduction). Small differences were observed across conditions, but they were not significantly different. This indicates the powered exoskeleton device was not largely affecting normal joint behavior, but instead it was substituting robotic torque and power for biological muscle torque and power. The one exception was that the powered exoskeleton did not allow full hip extension, which is difficult to prevent from a controls perspective.

One difference between the EMG controller and biological hip torque controller was torque generation in late swing. The biological hip torque control only began providing hip assistance at heel contact. The EMG controller started generating exoskeleton torque prior to heel contact. This generation of torque in late swing may have synced better with the human musculoskeletal system and aided some users in reducing metabolic cost. Notably, the overall mechanical power (Figure 10C) was larger in the case of the biological hip torque control compared to the EMG controller. Thus, the differences in metabolic cost were likely due to the timing of the hip actuation rather than the total amount of mechanical power delivered.

The state machine controller was able to deliver a profile during stance phase that closely emulated a biological torque profile. As compared to Winter’s data (Neumann, 2010), the state machine closely matched a human’s joint torque profile for hip flexion assistance but at a reduced magnitude. Hip extension assistance peak torque was slightly delayed but was still close to the physiological profile. During early swing phase, the exoskeleton was unpowered and demonstrated some drag (hip extension torque). It did not provide any hip extension assistance in late swing. The hip flexion assistance was very different between controllers. The state machine matched biological torque levels, but some subjects felt like it was aggressive and occurred too early to be useful. In contrast, the EMG control tended to feel like it was timed more appropriately and easier for subjects to adopt.

It is difficult to directly compare our results to those from previous experimental studies on hip exoskeletons, but there are some important considerations that emerge in light of both. Outcome measures of performance are a function of both the control architecture and the exoskeleton hardware, as well as subject factors such as physical capability and experience of the users. Ding et al. (2016a) delivered hip extension torque through an exoskeleton with a peak at ~15–20% of the gait cycle. Their metabolic cost reduction was between 5.7 and 8.5% compared to their unpowered condition. In comparison to our biological hip torque controller, which provided roughly two-thirds the level of assistance as in Ding et al. (2016a), both the timing of peak hip extension torque (~15% of the gait cycle) and the associated metabolic cost reduction (7.0% compared to unpowered) were comparable. A number of previous autonomous hip exoskeleton studies that have provided hip extension assistance have set their controllers to provide peak assistance well after heel contact. For example, in Seo et al. (2016) the timing of peak torque assistance was at 15% of the gait cycle, in Giovacchini et al. (2014) it was at ~25%, and in Sugar et al. (2017) it was at ~30% of the gait cycle. These studies use oscillator-based control to relate hip angle (as a phase variable) to joint torque. Another autonomous hip exoskeleton study by Seo et al. (2016) used a similar assistive profile as our biological torque controller, but at approximately 2.5 times the magnitude. Seo et al. found a substantial metabolic cost reduction of 13% compared to walking without an exoskeleton.

One of the primary differences between the EMG controller and the biological torque controller in our study was an earlier onset of hip extension for the EMG controller condition. Using EMG control, subjects received hip extension assistance with the testbed system before heel contact, and it peaked immediately after (~5%) heel contact. Our study found that providing hip extension earlier than most hip exoskeleton studies have done may be beneficial for the goal of reducing metabolic cost. These results suggest that autonomous exoskeleton designers may find it valuable to provide earlier hip extension assistance than suggested in previous literature studies. This interpretation is complicated due to the confounding factor of hip flexion assistance (which was also different between the two conditions), but a follow-up study we have conducted which tested only hip extension has verified this finding (Young et al., 2017). EMG may also be valuable to help provide more targeted assistance with stride-to-stride variability that controllers based on mechanical sensor feedback alone may have a more difficult time adjusting to in real time.

### Subject Considerations

We expected subjects to slowly adapt to the powered exoskeleton and reduce their metabolic rate over time, similar to previous experiments with EMG control with ankle exoskeletons (Sawicki and Ferris, 2009). However, our subjects showed very little change in metabolic cost over time in the powered conditions with the exoskeleton. With biological hip torque control, subjects did not significantly decrease their metabolic cost after the 10-min time point. With EMG control, there were no differences in time over the course of the 30-min walking trial. In less than 5 min, subjects adapted to the powered exoskeleton and chose a walking strategy that did not change appreciably over time.

Subjects’ perception of the exoskeleton varied greatly, but common trends emerged. All subjects felt that the two controllers were dramatically different. Some preferred the biological hip torque control while others preferred EMG control, and this did not necessarily match with the controller that had the lower metabolic cost for the subject (Figure 6). Instead, some subjects appreciated the regularity of the state machine in that each step felt very nearly the same. Most subjects felt that the EMG control had better timing and was more assistive, but subjects also consistently commented on the EMG control as less predictable due to the timing and the magnitude of the assistance potentially changing from step to step. This bothered some subjects more than others and was the primary reason as to why some subjects preferred biological hip torque control to EMG control. However, the majority of subjects preferred EMG control because they felt that it moved with them compared to the biological hip torque control that often felt like it was forcing them to walk in a particular manner.

### Limitations

Errors in the state machine were rare, but certain errors caused subjects to feel like it was a misstep due to the magnitude of the perturbation. We identified and quantified four different errors that occurred due to the state machine. Two types of errors were largely unfelt by the exoskeleton users. These errors associated with incorrect timing of the trigger between early and mid stance caused the hip extension torque to turn off too early or too late, which was mostly inconsequential. However, the errors in triggering late stance too early or too late were typically felt by the user. If late state was triggered too early, the hip flexion torque would come on too early, and users felt a small perturbation. If the late stance was too late, the hip flexion assistance was delayed or did not occur at all. This was the largest perturbation as users were accustomed to a strong hip flexion assistance in late stance and removing this assistance caused a mild misstep. Overall these errors were very rare as the state machine was highly accurate as less than 1% of the total steps taken had an error. Users actively perceived only a small fraction of these errors, but this may have slightly reduced the overall effectiveness of using a state machine for control though given the low error rate this should have had a negligible effect on metabolic cost.

One limitation of this study was that it was only conducted at a single speed and over steady state level-ground walking. The results could be considerably different at other speeds or varying terrains. Both EMG and biological hip torque control are capable of appropriately adapting to the user’s speed, but we did not test their capability to do so. A faster walking speed, closer to the optimal walking speed for metabolic cost of transport, would be an interesting case to study for able-bodied subjects. Similarly, a lower walking speed similar to most pathological walking speeds would be worthwhile to study as hip exoskeletons may be of some assistance for various pathological conditions.

The mechanical properties of the pneumatic actuators were another limitation because they prevented generation of a precise torque profile for the exoskeleton. Though it formed a close approximation, the state machine controller could not precisely replicate biological torque profiles due to the low force bandwidth of the actuators. Due to the actuator dynamics, there was a delay between control signal generation and torque generated in the exoskeleton (Figures 9 and 10). In addition, the pneumatic system tethered the exoskeleton such that we could not test it in over ground walking, which often varies from treadmill walking (Alton et al., 1998). A replacement of the pneumatic actuators with electromechanical actuators would potentially remove both of these limitations. Also, the pneumatic actuators had some frontal plane coupling with the sagittal plane which was an artifact of the mechanical lever used to actuate the device. This may have altered normal biomechanics to some extent and led to the larger step width observed in most subjects. Subjects may also have increased step width to avoid rubbing the thigh cuff of the exoskeleton, or because we instructed them to keep their feet separated on the two belts of the treadmill to obtain accurate force plate information.

Another limitation of the exoskeleton was the soft tissue interface. We had to provide a large amount of padding to maintain subject comfort over the course of the experiment. Between this soft padding and a subject’s soft tissue, which can be considerable in the thigh area, there was energy loss in the transfer from exoskeleton to human. In addition, the weight of the exoskeleton was substantial (6.8 kg) and caused a weight penalty in metabolic cost. Based on comparison to literature values of humans walking without an exoskeleton (Collins et al., 2015), it is likely that the weight penalty incurred by the exoskeleton (as measured by the metabolic cost in the unpowered condition) is similar to the metabolic cost reduction of the EMG control condition.

Finally, the rectus femoris was not an ideal muscle to use for myoelectric control of hip flexion. We effectively removed the contribution of the muscle toward knee extension in early stance with our controller, but there was relatively large intersubject variability with the rectus femoris compared to the other muscles that we measured. This caused slightly different controllers from subject to subject. However, even with the limitation presented it is the only hip flexor easily accessible with surface EMG and is likely the best choice for myoelectric control over hip flexion.

## Conclusion

The main objective of this study was to test two control strategies for providing hip assistance through a pneumatically powered exoskeleton during walking. We achieved this goal, demonstrating that our proportional myoelectric controller had superior performance in overall metabolic energy savings and muscle activity compared to a controller that targeted a biological torque profile. Subjects perceived that the EMG control in this experiment tended to feel smoother but appreciated the regularity provided by the biological torque controller. Future studies over a variety of terrains, robotic devices, and locomotion speeds are needed to fully reveal the potential of myoelectric control as a controller for robotic lower limb exoskeletons intended to decrease locomotion energetics.

## Ethics Statement

This study was approved by the University of Michigan Institutional Review Board under study number HUM00070022. All subjects provided written consent after reading the IRB approved consent document. They were verbally led through the document and given the opportunity to raise any questions or concerns about the protocol. N/A to this study.

## Author Contributions

AY helped in conceiving the study concept and design, acquiring the data, analyzing and interpreting the data, and drafting the manuscript. HG helped in acquiring the data, analyzing and interpreting the data, and drafting the manuscript. DF helped in conceiving the study concept and design, drafting and revising the manuscript, obtaining funding, and supervising the study.

## Conflict of Interest Statement

The authors declare that the research was conducted in the absence of any commercial or financial relationships that could be construed as a potential conflict of interest.

## Supplementary Material

Video S1 Demonstration of exoskeleton controllers.

### Hip Exoskeleton Controllers

The subject demonstrates using both the state machine control and the EMG-based control. This video demonstrates both the controllers used in this study.
